# The cerebellum plays more than one role in the dysregulation of appetite: Review of structural evidence from typical and eating disorder populations

**DOI:** 10.1002/brb3.3286

**Published:** 2023-10-13

**Authors:** Michelle Sader, Gordon D. Waiter, Justin H. G. Williams

**Affiliations:** ^1^ Biomedical Imaging Centre University of Aberdeen Aberdeen UK; ^2^ School of Medicine Griffith University Gold Coast Queensland Australia; ^3^ Gold Coast Mental Health and Specialist Services Gold Coast Queensland Australia

**Keywords:** anorexia nervosa, appetite regulation, cerebellum, eating disorders, meta‐analysis, obesity, review, voxel‐based morphometry

## Abstract

**Objective:**

Dysregulated appetite control is characteristic of anorexia nervosa (AN), bulimia nervosa (BN), and obesity (OB). Studies using a broad range of methods suggest the cerebellum plays an important role in aspects of weight and appetite control, and is implicated in both AN and OB by reports of aberrant gray matter volume (GMV) compared to nonclinical populations. As functions of the cerebellum are anatomically segregated, specific localization of aberrant anatomy may indicate the mechanisms of its relationship with weight and appetite in different states. We sought to determine if there were consistencies in regions of cerebellar GMV changes in AN/BN and OB, as well as across normative (NOR) variation.

**Method:**

Systematic review and meta‐analysis using GingerALE.

**Results:**

Twenty‐six publications were identified as either case–control studies (*n*_OB_ = 277; *n*_AN/BN_ = 510) or regressed weight from NOR data against brain volume (total *n* = 3830). AN/BN and OB analyses both showed consistently decreased GMV within Crus I and Lobule VI, but volume reduction was bilateral for AN/BN and unilateral for OB. Analysis of the NOR data set identified a cluster in right posterior lobe that overlapped with AN/BN cerebellar reduction. Sensitivity analyses indicated robust repeatability for NOR and AN/BN cohorts, but found OB‐specific heterogeneity.

**Discussion:**

Findings suggest that more than one area of the cerebellum is involved in control of eating behavior and may be differentially affected in normal variation and pathological conditions. Specifically, we hypothesize an association with sensorimotor and emotional learning via Lobule VI in AN/BN, and executive function via Crus I in OB.

## Graphical abstract

This meta‐analysis reviewed cerebellar volume in 26 publications comprising of case–control and normative cohorts. Findings identified differential reduction of the cerebellum across conditions, suggesting multiple cerebellar regions are implicated in the dysregulation of appetite or feeding behavior.

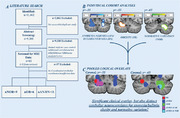

## 1 INTRODUCTION

### 1.1 Importance of studying appetite control mechanisms

Appetite control has a complex and multifaceted nature, and problems with eating behavior arise from a variety of genetic, cognitive, emotional, and physiological factors. Irregular appetite patterns result in abnormal body weight for height (indexed by the body mass index [BMI]) as well as irregular metabolic/mental health (Alhussain et al., 2016; Buyukkurt et al., 2021; Farshchi et al., 2005). For instance, obesity (OB), characterized by a BMI of >30, is a major public health concern pertaining to appetite dysregulation that is on the increase and constitutes as a major risk factor for conditions such as hypertension, diabetes, cardiovascular diseases, and cancer (Abell et al., 2007; Arroyo‐Johnson & Mincey, 2016; Zhang & Rodriguez‐Monguio, 2012). In the past 35 years, worldwide OB prevalence rates have nearly doubled, with 13% classifying with OB (WHO, 2014). At the opposite end of the BMI scale are individuals with anorexia nervosa (AN) who refrain from eating and harbor pathological fears of weight gain and food consumption (APA, 2013). AN is a complex multidimensional eating disorder characterized by pathologically decreased weight‐for‐age/height, with lifetime prevalence as high as 4% (Smink et al., 2013). Recently, publications found an increase in reported AN cases over time, although increased incidence may correlate with increased specificity of reporting protocols (Erskine et al., 2016; Sweeting et al., 2015). AN is relatively uncommon compared to other psychiatric disorders, yet mortality rates are greater, reporting between 2% and 6% (Arcelus et al., 2011; Wakeling, 1996). While AN and OB may not technically be eating disorders at opposite ends of a singular spectrum, there are prominent neuroanatomical (Allen et al., 2005; Amianto, D'Agata, et al., 2013; Augustijn et al., 2019; Boghi et al., 2011; Brooks et al., 2011; García‐García et al., 2019; Kakoschke et al., 2019; Leggio & Olivito, 2018; Martín‐Pérez et al., 2019; Milos et al., 2021; Nagahara et al., 2014; Sanders et al., 2015), metabolic, and genetic (Bulik et al., 2019; Chen et al., 2020; Fawcett & Barroso, 2010; Watson et al., 2019; Yang et al., 2017) factors linking both disorders. It is therefore of significance for research to identify mechanisms and neuroanatomical structures regulating appetite or weight, which may serve as treatment targets.

Despite these priorities, roles of the cerebellum (displayed in Figure S1; Diedrichsen & Zotow, 2015) in body weight or appetite control (Mendoza et al., 2010; Tuulari et al., 2016; Zhu & Wang, 2008) receive surprisingly little attention in appetite‐related research, despite their consistent implication. Traditionally, the cerebellum was thought to solely serve motor coordination and somatic functions, but further investigation reveals that this brain region plays a variety of diverse roles. Researchers have reported the cerebellum demonstrates organizational similarity to that of the cerebral cortex (Herrup, 2000) with anatomically segregated functions. Cerebellar contributions to intrinsic connectivity networks have shown that particular regions of the cerebellum are distinctly involved in different cognitive functions (Habas et al., 2009; Stoodley, 2012; Stoodley & Schmahmann, 2009) and implicated in five intrinsic connectivity networks, including the executive control network (ECN; via Lobule VIIB; Crus I/II), default‐mode network (via Lobule IX), salience network (via Lobule VI), and sensorimotor network (via Lobule VI) (Habas et al., 2009). Contrasting traditional conceptualization, motor tasks are largely consigned to Lobule VIIIa/b and represent limited cerebellar functionality (Stoodley & Schmahmann, 2009). Current views of the cerebellum implicate it in homeostatic regulation (Saker et al., 2014; Supple Jr. & Kapp, 1993), executive/cognitive functionality (Buckner, 2013; Contreras‐Rodríguez et al., 2017; D‘Angelo & Casali, 2013; Habas et al., 2009; Koziol et al., 2014; Leiner et al., 1986; Stoodley & Schmahmann, 2009; Stoodley et al., 2012) (including habit formation [D‘Angelo & Casali, 2013], conditioning behaviors [D‘Angelo & Casali, 2013; Dolan, 1998; Habas et al., 2009, 2013; Molinari et al., 2008; Utz et al., 2015], procedural knowledge storage [Dolan, 1998; Habas et al., 2009, 2013; Zhu et al., 2011], working memory function [Molinari et al., 2008; Stoodley & Schmahmann, 2009], and cravings [Carnell et al., 2014]), and emotional regulation (Adamaszek et al., 2017; Baumann & Mattingley, 2012; Buckner, 2013; Habas et al., 2009, 2013; Lupo et al., 2015; Schmahmann & Pandya, 1997; Shobe, 2014; Stoodley & Schmahmann, 2009; Utz et al., 2015). Importantly, evidence suggests the cerebellum may participate in aspects of food intake and appetite control through multiple mechanisms, including physiological (i.e., feeding circuit connectivity, response/influence on gut hormones/neurotransmitters), cognitive (i.e., food palatability, feeding‐related memories), and emotional (i.e., food‐related cravings) means (Cavdar et al., 2001; Wright et al., 2016).

### 1.2 A cerebellar role in weight and appetite regulation

On a physiological scale, the cerebellum interacts via extensive signaling networks with the hypothalamus and insula, which both contain networks specific to food intake (Cavdar et al., 2001; Contreras‐Rodríguez et al., 2017) via neural and hormonal mechanisms (Allen et al., 2005; Contreras‐Rodríguez et al., 2017; Zhao et al., 2017). Enteric nervous system gut hormones, such as leptin and ghrelin, interactively modulate regions of the brain associated with food intake control including the cerebellum, hypothalamus, and brainstem (Bouret et al., 2004; Hommel et al., 2006). In response to ghrelin, cerebellar activation decreases and likely works to stimulate appetite via ghrelin‐induced suppression of satiety hormones, such as cholecystokinin, within vagal afferent neurons (Al Massadi et al., 2017; Gavello et al., 2016; Jones et al., 2012). Recently, Choe et al. (2021) investigated synchrony between basal gastric rhythm (governing both vagal activity and peristalsis) and brain activity, identifying the strongest phase‐locked synchrony within the cerebellum (Choe et al., 2021) that suggests a more direct relationship between gastric mechanisms and cerebellar functionality.

The cerebellum is also implicated in genetic aspects associated with body weight disorders. Beyond neuroanatomical/biological aspects of appetite dysregulation, disorders on the extremes of the standard BMI measure (i.e., AN/OB) exhibit shared genetic and metabolic correlations (Chen et al., 2020; Fawcett & Barroso, 2010; Watson et al., 2019; Yang et al., 2017). With genetic associations occurring in opposing directions, they have been termed metabolic “mirror images” of one another (Bulik et al., 2019). Cerebellar tissues and pathways have recently been implicated in aspects of genetic risk for both conditions (Cheng et al., 2020; Watson et al., 2019). Evidence from AN‐ and cerebellum‐related studies suggests that deficits in cerebellar mRNA expression occur in fetal and early‐life AN pathogenesis (Cheng et al., 2020), and altered cerebellar volume may explain body image disturbances in AN (Briatore et al., 2020; Gaudio et al., 2011; Leibovitz et al., 2018; Watson et al., 2019). In OB, a multitude of genes have been associated with increased risk, predominantly the fat mass (FTO) and melanocortin‐4 receptor genes. FTO is expressed in regions such as the hypothalamus, hippocampus, and cerebellum (Madsen et al., 2009; McTaggart et al., 2011; Miller et al., 2009; Wang et al., 2012), and research suggests it is highly associated with OB outcome (Fawcett & Barroso, 2010; Yang et al., 2017).

The cerebellum is also repeatedly reported to participate in neurobiological modulation of dopaminergic and serotonergic signaling (Cutando et al., 2022; Oostland & Hooft, 2016), which becomes dysregulated in conditions of abnormal weight/BMI (Berridge et al., 2010; Bohon, 2017; Carter et al., 2016; Kawakami et al., 2022; Kaye et al., 2013; van Galen et al., 2018). Dopaminergic‐related mechanisms driving both OB and AN have also been associated with addiction‐resembling behavior related to eating behavior (Barbarich‐Marsteller et al., 2011; Berridge, 2009; O'Hara et al., 2015). Recently, Low et al. (2021) used a reverse‐translational approach to identify a cerebellar‐based satiation network. In humans, food cues activate cerebellar output neurons to promote satiation through reductions in phasic dopaminergic responses to food. As such, the cerebellum is likely implicated in altered neurobiological mechanisms associated with dysregulated appetite or weight, such as those with AN/OB.

Importantly, extant literature also suggests that the cerebellum may participate in more conscious domains of appetite, such as emotional and cognitive aspects driving appetitive behaviors. The cerebellum may play a substantive role in cognitive aspects of appetite control via an individuals’ subjective feeling of craving or outcome expectation built off previous experience and memory (Carnell et al., 2014; Garrison et al., 2016; Geliebter et al., 2016; Moreno‐Rius & Miquel, 2017; Noori et al., 2016; Tomasi et al., 2015). The cerebellum is also associated with traits of impulsivity and loss‐of‐control (LOC) eating. Recent investigations also unveiled cerebellar roles in the portion size effect (PSE) (Herman et al., 2015; Rolls et al., 2006; Steenhuis & Poelman, 2017), the phenomenon where more is eaten when large quantities of food are available (English et al., 2019).

### 1.3 Cerebellar volume and dysregulation of appetite

Structural associations between the cerebellum and conditions of dysregulated BMI and appetite have been consistently documented. This includes evidence from loss‐of‐function studies (Zhu & Wang, 2008), as well as animal studies where lesions or removal of cerebellar hemispheres leads to reduced appetite, pathological weight loss, and increased mortality rate (Colombel et al., 2002). In humans, Oya et al. (2014) report a high proportion of cerebellar tumor detection with associated AN. Similarly, cerebellar degeneration associated with ataxia correlates with increased likelihood of being underweight or experiencing abnormal appetite (Kronemer et al., 2021; Rönnefarth et al., 2020; Ross et al., 2015; Sánchez‐Kuhn et al., 2017). Due to appetite disturbances emerging post‐cerebellar structural abnormalities, these studies indicate that cerebellar deficits could play a causative role in the loss of appetite.

Cerebellar volume has also been directly associated with conditions of under‐ and overeating. Excess weight (BMI > 25.5) or OB (BMI > 30.0) has been associated with both increased (Brooks et al., 2011) and decreased concentrations of gray matter volume (GMV) in the left (Brooks et al., 2011), bilateral (Kakoschke et al., 2019), and bilateral posterior (García‐García et al., 2019) cerebellum. Prefronto‐cerebellar circuits, implicated in cognition, emotion, executive function, and error detection, exhibit volume reduction in those with OB (Allen et al., 2005; Brooks et al., 2011). Recovery from OB is also associated with positive changes in cerebellar volume and is thought to be important in treatment as well as conditioning of eating behavior (Augustijn et al., 2019). Differences in cerebellar volume have been noted in both rat and human models of AN (Fagundo et al., 2012; Moyse et al., 2019; O'Hara et al., 2015). Within humans, cerebellar atrophy and cellular loss are associated with AN disease duration and poor treatment success (Milos et al., 2021), persist after weight recovery, and are suggested to play a role in maintaining a low body weight (Boghi et al., 2011; Nagahara et al., 2014). A multimodal meta‐analysis by Zhang et al. (2018) identified bilateral reduction of the cerebellum in those with AN, suggested to be associated with symptoms or traits of dietary restriction and appetitive inflexibility (Zhang et al., 2018). A systematic volumetric review by Seitz et al. (2018) also demonstrated that volumetric atrophy of the cerebellum upon assessment predicted AN outcome at 1‐year follow‐up.

Altogether, the cerebellum may function as a pivotal point of behavioral or associative tuning in relation to cognitive aspects of appetite regulation. Schmahmann et al. (2019) identified associations between the posterior cerebellar lobes and cognition, proposing “The Dysmetria of Thought” (DoT) theory. This theory suggests that, by receiving multimodal inputs and establishing procedurally optimized behavioral modulation, the cerebellum modulates cognition in a similar way to which it smooths and fine‐tunes coordination of motor behavior. In cases of DoT, the cerebellum may perform similarly regarding altered input and integration of appetite‐related stimuli, such as formation of rigid, repetitive, or inflexible individualized food‐related models, memories, and expectations formed from previous interactions with food stimuli. Cerebellar regions attending to salience of stimuli, such as Lobule VI, may be associated with body weight/appetite changes via formations of negative associations with food that could contribute toward dietary restriction or rigidity. Alternatively, cognitive/executive abnormalities reported in those with OB may suggest associations with cerebellar regions prominent to the ECN, such as Crus I, where the reward value or experiential engagement with food stimuli may be too “positively” integrated, contributing toward repetitive engagement with stimuli to prolong or increase the frequency of a rewarding experience.

### 1.4 Aims of study

Evidence provided by wide‐ranging cerebellar research demonstrates that the cerebellum is important in modulating aspects of appetite control and implicated in both OB and AN. However, whether identical or differing areas of the cerebellum are associated with AN and OB is unclear. This systematic review aimed to examine cerebellar anatomy reported in case–control AN and OB literature, as well as across the weight range via normative (NOR)/nonclinical populations to investigate whether cerebellar structure differs across body weight states and disorders. We generated two alternative hypotheses: We first hypothesized that a singular area of the cerebellum, such as Crus I (implicated in executive control circuits [Augustijn et al., 2019]), would be associated with abnormal BMI conditions, and that respective regions affected in OB and AN would be similar. Our alternative, competing hypothesis was that separate cerebellar regions would be altered across conditions and shown in distinct regions. For instance, Crus I volume could be primarily affected in OB, while Lobule VI volume (implicated in salience/sensorimotor circuits) could be primarily affected in AN.

## 2 METHODOLOGY

### 2.1 Selection of literature

Literature was searched on June 29, 2022 using SCOPUS, PubMed Central (PMC), and Web of Science (WoS) using identical search criteria. SCOPUS identified 2638 publications involving cerebellar volume during OB—search criteria: (cerebell* AND obesity AND MRI) and 572 publications regarding AN (cerebell* AND anorexia AND MRI). PMC identified 4950 publications relating to OB, as well as 461 papers regarding AN. Lastly, 2588 and 233 papers were identified through WoS regarding cerebellar characteristics in those with OB and AN, respectively. Inclusion criteria involved presence of key phrases utilized in search, including “gray literature,” or findings produced outside of traditional publishing. Exclusion criteria were as follows: (1) publications older than 10 years, as CB imaging methods have significantly improved since 2010 (Gutierrez et al., 2014; Sirin et al., 2005); (2) animal studies; (3) publications not reporting case–control studies; (4) inclusion of clinical groups/correlations unrelated to the study scope; (5) publications not utilizing voxel‐based morphometry (i.e., diffusion tensor imaging/functional MRI [fMRI] studies); and (6) publications with results not reported in Talairach/MNI coordinates. Individual studies fitting criteria were additionally collected from meta‐analyses (*n* = 3).

Two publications (Huang et al., 2019; Frank et al., 2013) could not be further assessed as findings consisted of increased cerebellar volume/positive correlations, and insufficient positive coordinate‐based data were available across cohorts to generate meta‐analytic positive CB findings. Three AN publications (Joos et al., 2010; Amianto, Caroppo, et al., 2013; D'Agata et al., 2015) included individuals with bulimia nervosa (BN) that contributed to AN findings. As both conditions report with significant diagnostic crossover, with 34.1% of AN individuals experiencing crossover to BN over a 7‐year follow‐up (Eddy et al., 2008), these papers were included to form the AN/BN cohort. Due to the limited literature, we were unable to correct for age and gender across data sets.

**FIGURE 1:**
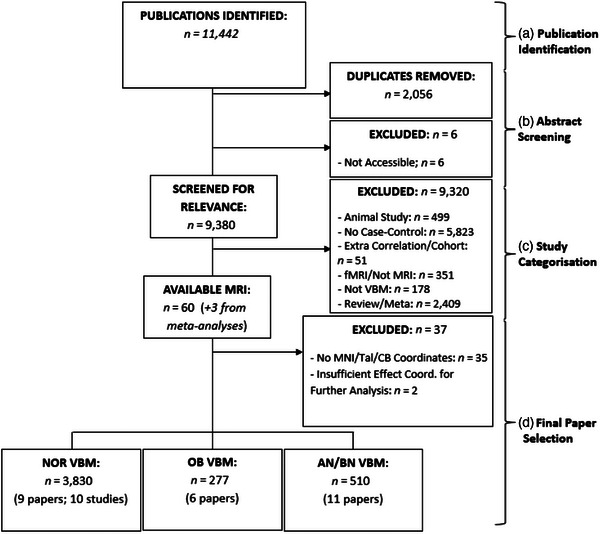
Flowchart depicting the identification (a), abstract screening (b), categorization (c), and final selection (d) of papers. Three papers were also found from meta‐analyses (Dommes et al., 2013; Huang et al., 2019; Weise et al., 2019). AN, anorexia nervosa; BN, bulimia nervosa; CB, cerebellum; Coord., coordinates; fMRI, functional magnetic resonance imaging; MNI, Montreal Neurological Institute; MRI, magnetic resonance imaging; NOR, normative; OB, obesity; Tal, Talairach; VBM, voxel‐based morphometry.

In summary, six OB (Dommes et al., 2013; Jauch‐Chara et al., 2015; Mueller et al., 2012; Ou et al., 2015; Shan et al., 2019; Wang et al., 2017) and 11 AN/BN (Amianto, Caroppo, et al., 2013; Bomba et al., 2015; D'Agata et al., 2015; Fonville et al., 2014; Gaudio et al., 2011; Joos et al., 2010; Lenhart et al., 2022; Mishima et al., 2021; Phillipou et al., 2018) papers included coordinates and were selected for final analysis (participant numbers: *n*_case–control_ = 787; *n*_OB_ = 134 vs. *n*_HC_ = 143; *n*_AN_ = 228 and *n*_BN_ = 48 vs. *n*_HC_ = 234) (Table 1; Figure 1). As an exploratory assessment of condition‐specific effects, an AN‐only cohort was comprised by excluding three previously included AN papers (Amianto, Caroppo, et al., 2013; D'Agata et al., 2015; Joos et al., 2010) evaluating those with BN in conjunction with AN (eight papers; *n*_AN_ = 178 vs. *n*_HC_ = 185).

**TABLE 1:** Sociodemographic and statistical data for included studies across AN/BN (*n* = 11), OB (*n* = 6), and NOR (*n* = 10) cohorts.

First author	Number of subjects				Age, years (mean ± *SD*)				BMI (mean ± *SD*)			DOI, M. (mean ± *SD*)		Correction type	
AN/BN^a^	*n*_AN_	*n*_HC_	*n*_BN_	M:F ratio	AN	HC	BN	A^b^	AN	HC	BN	AN	BN	FWE^c^	Threshold
Lenhart, 2022	22	18	0	0:40	15.2 ± 1.2	16.8 ± 0.9	N/A	0	15.4 ± 1.4	21.2 ± 1.0	N/A	9.4 ± 6.8	N/A	1	*p* < .05
Mishima, 2021	35	35	0	0:70	36.3 ± 10.0	36.0 ± 9.6	N/A	1	14.2 ± 2.5	21.0 ± 2.9	N/A	188.4 ± 108.0	N/A	1	*p* < .05
Phillipou, 2018	26	27	0	0:53	22.8 ± 6.7	22.5 ± 3.2	N/A	1	16.6 ± 1.2	22.6 ± 3.5	N/A	77.0 ± 89.2	N/A	1	1.68
D'Agata, 2015^a^	21	17	18	0:56	21.0 ± 5.0	22.0 ± 5.0	23.0 ± 4.0	1	16.1 ± 0.9	21.5 ± 2.3	22.0 ± 2.3	>24.0	>24.0	1	1.71
Fonville, 2014	33	33	0	N/A	23.0 ± 10.0	25.0 ± 4.0	N/A	1	15.8 ± 1.4	21.8 ± 1.8	N/A	84.0 ± 120.0	N/A	1	1.67
Amianto, Caroppo, et al., 2013, ^a^	17	14	13	0:44	20.0 ± 4.0	24.0 ± 3.0	22.0 ± 3.0	1	16.0 ± 1.0	21.0 ± 2.0	22.0 ± 2.0	13.0 ± 8.0	10.0 ± 5.0	1	1.7
Bomba, 2013	11	8	0	0:19	13.6 ± 2.8	13.4 ± 2.4	N/A	0	12.8 ± 0.8	19.9 ± 1.5	N/A	14.5 ± 10.9	N/A	1	1.78
Brooks, 2011	14	21	0	0:35	26.0 ± 1.9	26.0 ± 2.1	N/A	1	15.6 ± 0.4	21.4 ± 0.5	N/A	110.4 ± 22.8	N/A	1	1.69
Boghi, 2011	21	27	0	0:48	29.0 ± 10.0	30.8 ± 8.7	N/A	1	15.5 ± 1.8	21.9 ± 1.5	N/A	11.3 ± 12.1	N/A	1	1.7
Gaudio, 2011	16	16	0	0:32	15.2 ± 1.7	15.1 ± 1.5	N/A	0	14.2 ± 1.4	20.2 ± 1.6	N/A	5.3 ± 3.2	N/A	0	*p* < .001
Joos, 2010^a^	12	18	17	0:47	25.0 ± 4.8	26.9 ± 5.8	24.5 ± 4.8	1	16.0 ± 1.2	21.1 ± 2.0	21.1 ± 2.5	56.4 ± 43.2	90.0 ± 68.4	0	3.1
OB	*n*_OB_	*n*_HC_	–	M:F ratio	OB	HC	–	A^b^	OB	HC	–	–	–	FWE^c^	Threshold
Shan, 2019	37	39	–	41:35	27.8 ± 6.9	26.7 ± 6.8	–	1	40.0 ± 6.5	21.8 ± 1.8	–	–	–	1	1.67
Wang, 2017	31	49	–	13:7	39.6 ± 1.9	29.6 ± 1.4	–	1	34.4 ± 0.7	21.9 ± 0.3	–	–	–	0	2.64
Ou, 2015	12	12	–	1:1	9.1 ± 0.9	9.8 ± 0.7	–	0	24.4 ± 3.4	5.8 ± 1.0	–	–	–	1	1.73
Jauch‐Chara, 2015	15	15	–	30:0	24.7 ± 0.7	24.6 ± 0.7	–	1	36.3 ± 1.0	23.2 ± 0.4	–	–	–	1	1.7
Dommes, 2013	12	12	–	0:24	29.3 ± 7.3	27.5 ± 4.3	–	1	36.3 ± 4.8	20.9 ± 1.7	–	–	–	1 (FDR)	*p* < .05
Mueller, 2012	27	16	–	22:21	26.4 ± 5.4	24.8 ± 3.0	–	1	33.0 ± 6.4	22.5 ± 2.0	–	–	–	1	1.68
NOR	*n*_OB_	*n*_HC_	–	M:F ratio	OB	HC	–	A^b^	OB	HC	BMI range		–	FWE^c^	Threshold
Weise, 2019 (Cohort^a^)	0	136	–	11:23	N/A	29.6 ± 3.2	–	1	N/A	27.5 ± 4.9	N/A	–	–	1	1.66
Weise, 2019 (Cohort ^b^)	0	306	–	20:31	N/A	29.4 ± 3.4	–	1	N/A	26.3 ± 4.7	N/A	–	–	1	1.65
Huang, 2019	0	653	–	204:449	N/A	19.5 ± 1.5	–	1	N/A	20.8 ± 2.7	15.4–34	–	–	1	*p* < .05
Yao, 2016	0	109	–	62:47	N/A	35.2 ± 11.2	–	1	N/A	27.6 ± 6.1	<18‐>30	–	–	0	*p* = .001
Figley, 2016	0	32	–	1:1	N/A	26.2 ± 4.4; 23.5 ± 4.2	–	1	N/A	N/A	<18–25	–	–	1	*p* < .05
Masouleh, 2016	0	617	–	359:258	N/A	68.7 ± 4.6	–	1	N/A	27.5 ± 4	16.8‐41.4	–	–	1	3.1
Janowitz, 2015	0	2344	–	1087:1257	N/A	49.8 ± 9.3; 46.3 ± 11.3	–	1	N/A	27.4 ± 4.5; 27.2 ± 4.4	N/A	–	–	1	1.65
Kurth, 2013	0	115	–	54:61	N/A	45.2 ± 15.5	–	1	N/A	25.0 ± 4.1	18.2–42.3	–	–	1	1.66
Weise, 2013	0	76	–	13:6	N/A	32.1 ± 8.8	–	1	N/A	29.8 ± 8.9	<25‐>30	–	–	1	*p* < .05
Walther, 2010	0	95	–	0:95	N/A	69.3 ± 9.3	–	1	N/A	28.3 ± 2.1	<22‐>30	–	–	0	*p* < .001

Abbreviations: AN, anorexia nervosa; BMI, body mass index; BN, bulimia nervosa; FDR, false discovery rate; FWE, family‐wise error; HC, healthy control; M/F, male/female; OB, obesity.

^a^ Publications utilized in AN/BN cohort analysis, but excluded from exploratory AN‐only analysis.

^b^ Adult sample present: 1, yes; 0, no.

^c^ Correction for family‐wise error; 1, yes; 0, no.

While conducting the OB literature search, publications were identified evaluating correlations between BMI and GMV in nonclinical, NOR populations that fell in line with remaining inclusion criteria (*n* = 9 papers; 10 studies) (Figley et al., 2016; Janowitz et al., 2015; Kurth et al., 2013; Masouleh et al., 2016; Walther et al., 2010; Weise et al., 2019, 2013; Yao et al., 2016). As authors within the NOR subgroup predominantly conducted recruitment using community‐based methods and data repositories, participant aggregation generated larger sample sizes than the OB and AN/BN literature. These publications evaluated weight states across the BMI spectrum, with all but one (Figley et al., 2016) study (*n* = 8/9 papers) including individuals with excess weight, and all but three (Figley et al., 2016; Janowitz et al., 2015; Weise et al., 2019) studies (*n* = 6/9 papers) including those with OB. Papers were included in a separate NOR data set for analysis (participant number = 3830) for a total participant sample size of *n* = 4617.

### 2.2 Voxel‐based morphometry and ALE analysis

Cerebellar coordinates (values depicted in Table S1) were respectively incorporated into GingerALE, an activation likelihood estimate (ALE) meta‐analysis software using both Talairach and MNI space (Eickhoff et al., 2009). GingerALE converted Talairach coordinates to MNI space using the icbm2tal transform. The *p*‐value thresholds for individual analyses were conducted at the whole‐brain level and corrected for family‐wise error (FWE; .05) at 1000 permutations and set to *p* < .01. First, individual observations on respective AN/BN (*n* = 510) and OB (*n* = 277) cohorts were conducted, with a third analysis investigating negative correlations between BMI and cerebellar volume within an NOR data set (*n* = 3830). As an additional analysis, the AN data set was reassessed upon exclusion of publications (Amianto, Caroppo, et al., 2013; D'Agata et al., 2015; Joos et al., 2010) evaluating volumetric differences in BN (AN‐only) but not further investigated for overlap. Condition‐respective findings were assessed for overlap via logical overlays and conjunction analyses to visualize combinatorial clusters within AN/BN‐OB, AN/BN‐NOR, and OB‐NOR data sets. Jackknife analyses were conducted to assess robustness of findings via identical reassessment of cohort data upon one‐by‐one omission of publications. Additional information regarding cerebellar parcellation, subregion locations, and conjunction analysis thresholding is described within the Supporting Information.

## 3 RESULTS

### 3.1 Volumetric reduction in OB

**FIGURE 2:**
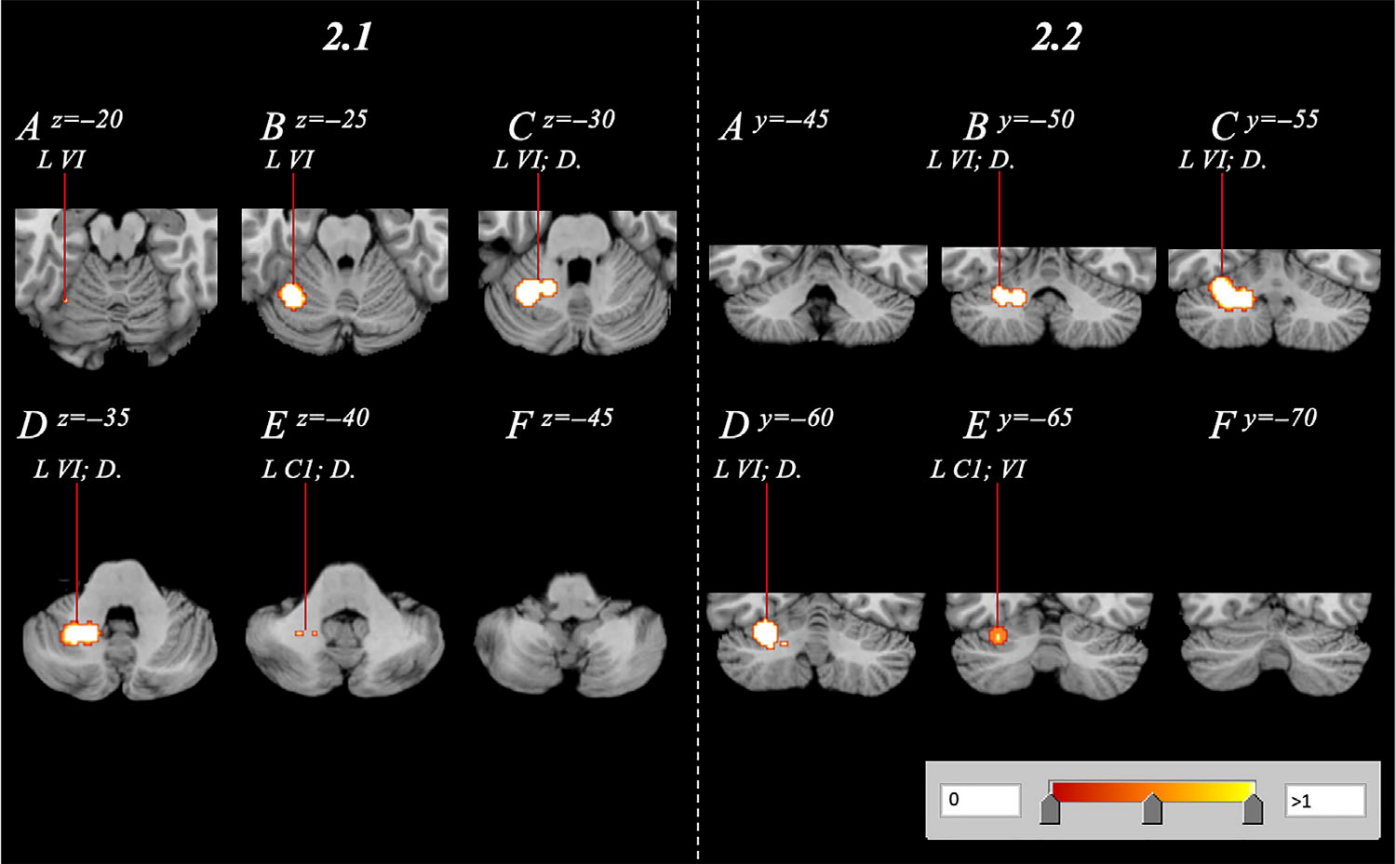
Cerebellar volume reduction of obesity subjects relative to healthy controls, spanning the right cerebellum with a range of *z* = −20 to −40 and *y* = −50 to −65 in both axial (2.1A–F) and coronal (2.2A–F) orientations. C1, Crus 1; D., dentate; L, left; R, right; VI, Lobule 6.

Within OB studies, volume of the cerebellum was found to be decreased in the left cerebellar hemisphere, with no bilateral effect. The cluster contained a volume of 3.58 cm^3^ with a cluster center of −27, −56, −31. Analysis revealed four peaks of significance partially anteriorly and posteriorly located (Table 2). Specific cerebellar regions affected are Lobule VI, Crus I, and the dentate gyrus (Figure 2).

**TABLE 2:** Coordinates of significance in OB studies (n = 6).

Region (OB < HC)	MNI coordinates			Volume (cm^3^)	ALE score	*p*	*Z*
	*x*	*y*	*z*				
L CB	–27	–56	–31	3.58	.00960	5.89 × 10^–5^	3.85
L CB, anterior L.	–32	–56	–28		.00956	5.89 × 10^–5^	3.85
L CB, anterior L.	–30	–60	–26		.00941	6.13 × 10^–5^	3.84
L CB, posterior L.	–28	–54	–34		.00920	1.36 × 10^–4^	3.64
L CB, posterior L.	–18	–54	–34		.00878	2.27 × 10^–4^	3.51

Abbreviations: ALE, activation likelihood estimation; CB, cerebellum; HC, healthy control; L, left; L., lobe; MNI, Montreal Neurological Institute; OB, obesity; R, right.

### 3.2 Volumetric reduction in AN/BN

**FIGURE 3:**
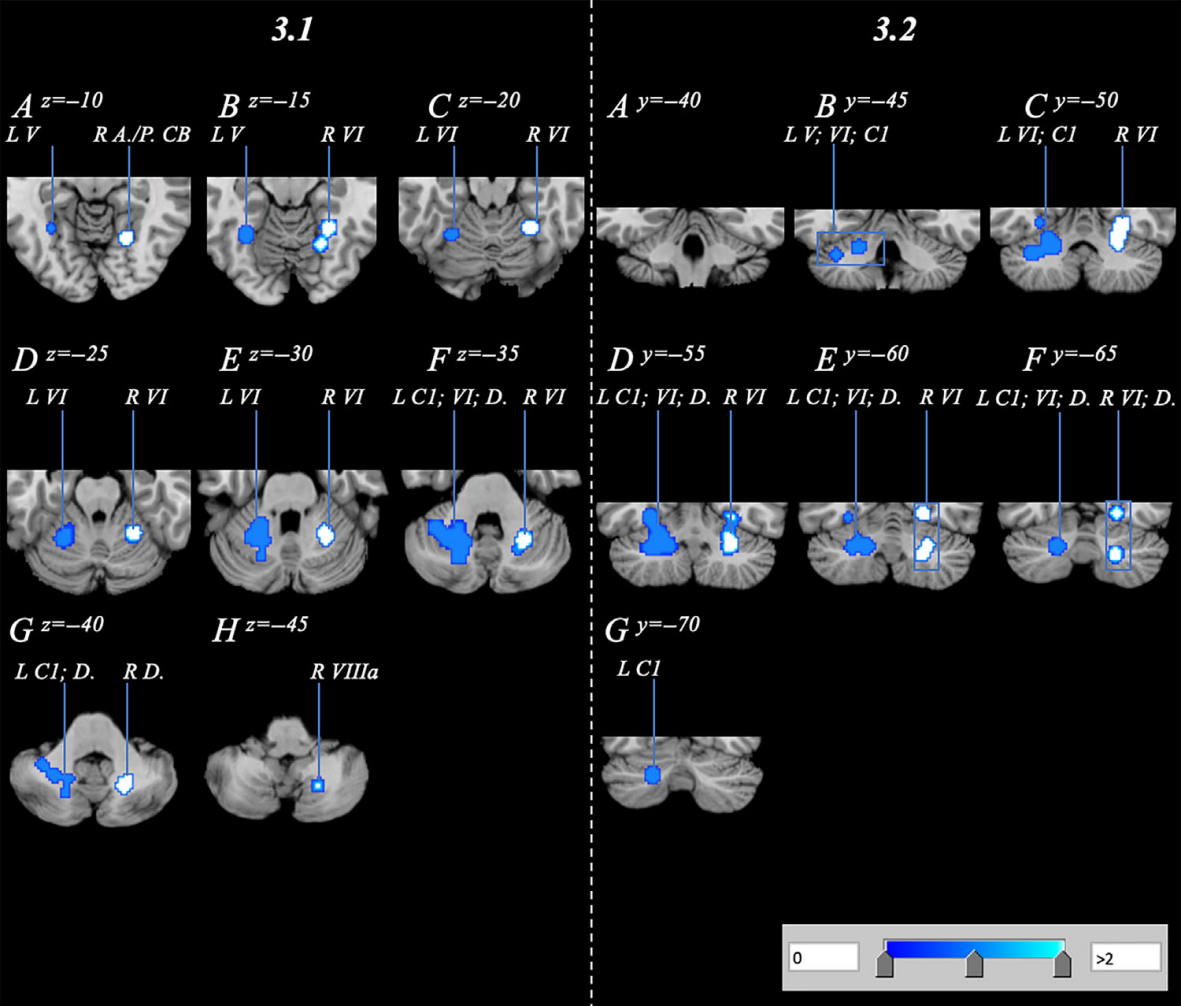
Cerebellar volume reduction of anorexia nervosa/bulimia nervosa subjects relative to healthy controls, spanning the bilateral cerebellum with a range of *z* = −10 to −45 and *y* = −45 to −70 in axial (3.1A–H) and coronal (3.2A–G) orientations. A., anterior; C1, Crus 1; CB, cerebellum; D., dentate; L, left; P., posterior; R, right; VI, Lobule 6; VIIIa, Lobule 8a.

The AN/BN GingerALE analysis revealed two significant clusters displaying bilateral reduction of cerebellar volume. The larger cluster was located within the left posterior lobe and contained a volume of 7.09 cm^3^ with a cluster center of −25, −55, −31. The region contained an additional seven peaks, with four of these occurring in the posterior lobe (Table 3). The smaller cluster comprising 4.56 cm^3^ was located in the right hemisphere, with three of five peaks located in the anterior lobe. Identified subregions included Crus I, the bilateral Lobule IV, and the bilateral dentate (Figure 3). Contrasting AN/BN findings, the exploratory AN‐only GingerALE analysis revealed no significant clusters displaying volumetric reduction within the cerebellum.

**TABLE 3:** Coordinates of significance in AN/BN studies (*n* = 11).

Region (AN/BN < HC)	MNI coordinates			Volume (cm^3^)	ALE score	*p*	*Z*
	*x*	*y*	*z*				
L CB	–25	–55	–31	7.09	.0106	2.10 × 10^–5^	4.10
L CB, anterior L.	–24	–54	–26		.0106	2.10 × 10^–5^	4.10
L CB, posterior L.	–28	–56	–36		.0105	2.18 × 10^–5^	4.09
L CB, posterior L.	–36	–48	–36		.00983	5.41 × 10^–5^	3.87
L CB, posterior L.	–18	–58	–34		.00973	6.88 × 10^–5^	3.81
L CB, anterior L.	–20	–48	–30		.00960	7.23 × 10^–5^	3.80
L CB, anterior L.	–30	–54	–16		.00923	1.37 × 10^–4^	3.64
L CB, posterior L.	–20	–68	–36		.00916	1.72 × 10^–4^	3.56
R CB	24	–56	–26	4.56	.0105	2.20 × 10^–5^	4.09
R CB, anterior L.	26	–56	–34		.0105	2.20 × 10^–5^	4.09
R CB, anterior L.	28	–50	–16		.0103	2.45 × 10^–5^	4.06
R CB, anterior L.	24	–50	–26		.0102	2.63 × 10^–5^	4.04
R CB, posterior L.	22	–62	–12		.00975	5.68 × 10^–5^	3.86
R CB, posterior L.	22	–62	–40		.00932	1.18 × 10^–4^	3.68

Abbreviations: ALE, activation likelihood estimation; AN, anorexia nervosa; BN, bulimia nervosa; CB, cerebellum; HC, healthy control; L, left; L., lobe; MNI, Montreal Neurological Institute; R, right.

### 3.3 NOR analysis: Cerebellum GMV versus BMI

**FIGURE 4:**
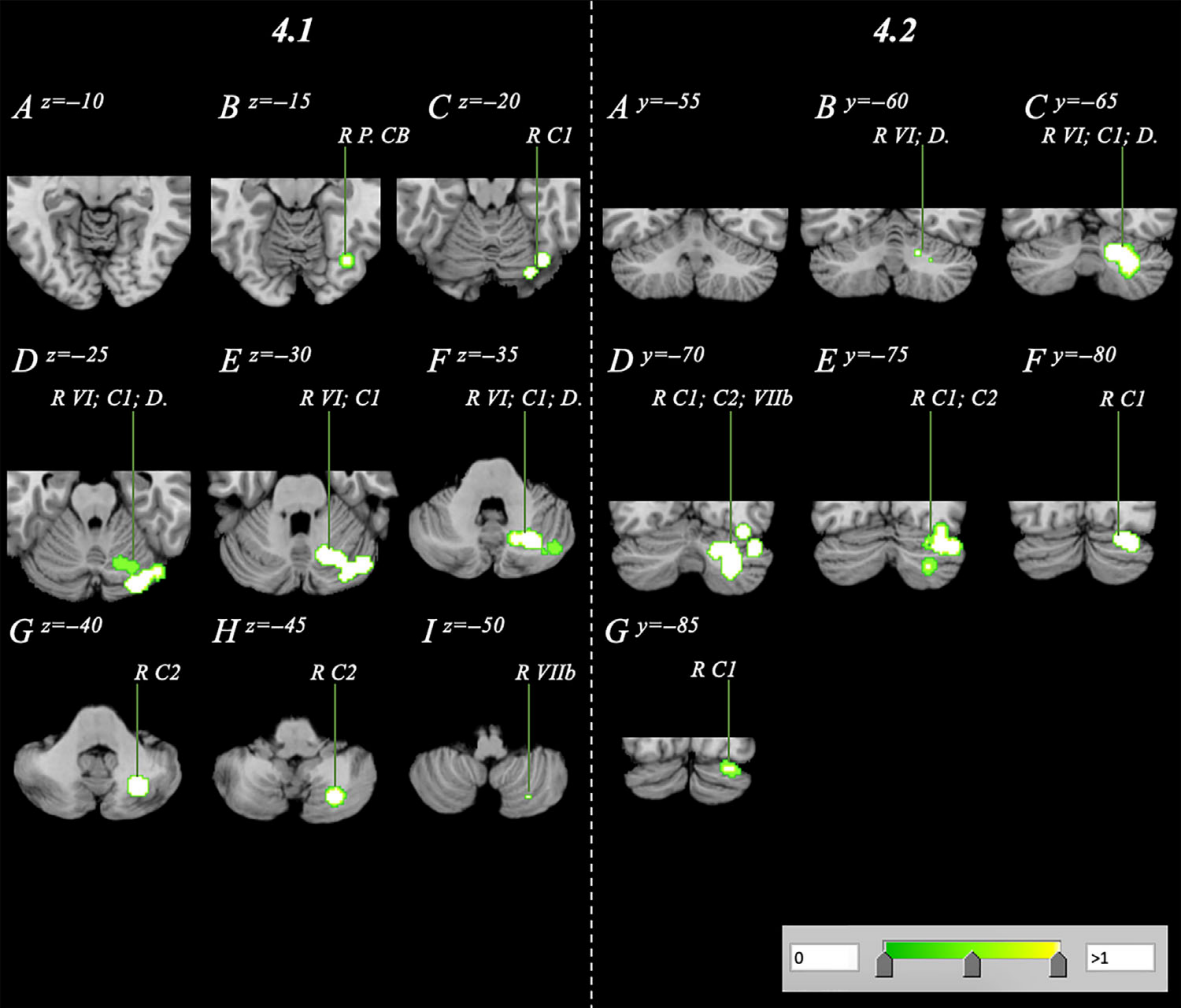
Volume reduction within a normative sample analysis spanning the right cerebellum with a range of *z* = −15 to −50 and *y* = 60 to −85 in axial (4.1A–I) and coronal (4.2A–G) orientations. C1, Crus 1; C2, Crus 2; D., dentate; L, left; P., posterior; R, right; VI, Lobule 6; VIIb, Lobule 7b; VIIIa/b, Lobule 8a/b.

Analysis of the NOR data set evaluating correlations between volumetric measures and BMI revealed a singular posterior cluster where cerebellum GMV negatively correlated with BMI. The cluster had a volume of 8.02 cm^3^ with a cluster center at 30, −71, −32 (Table 4; Figure 4). The cluster comprised seven peaks, all located within the posterior lobe affecting regions such as right Crus I/II, Lobule VI, Lobule VIIb, and the dentate.

**TABLE 4:** Clusters associated with increased BMI in NOR populations (*n* = 9/10).

Region (NOR; CB GMV vs. BMI)	MNI coordinates			Volume (cm^3^)	ALE score	*p*	*Z*
	*x*	*y*	*z*				
R CB	30	–71	–32	8.02	.0224	1.39 × 10^–8^	5.56
R CB, posterior L.	28	–70	–42		.0224	1.39 × 10^–8^	5.56
R CB, posterior L.	44	–72	–30		.0207	5.54 × 10^–8^	5.31
R CB, posterior L.	18	–66	–30		.0189	4.14 × 10^–7^	4.93
R CB, posterior L.	28	–80	–24		.0138	1.50 × 10^–5^	4.17
R CB, posterior L.	34	–78	–28		.0133	1.91 × 10^–5^	4.12
L CB, posterior L.	36	–72	–18		.011	6.86 × 10^–5^	3.81

Abbreviations: ALE, activation likelihood estimation; BMI, body mass index; CB, cerebellum; L, left; L., lobe; GMV, gray matter volume; MNI, Montreal Neurological Institute; NOR, normative; R, right.

### 3.4 Pooled volumetric reduction and conjunction analyses

**FIGURE 5:**
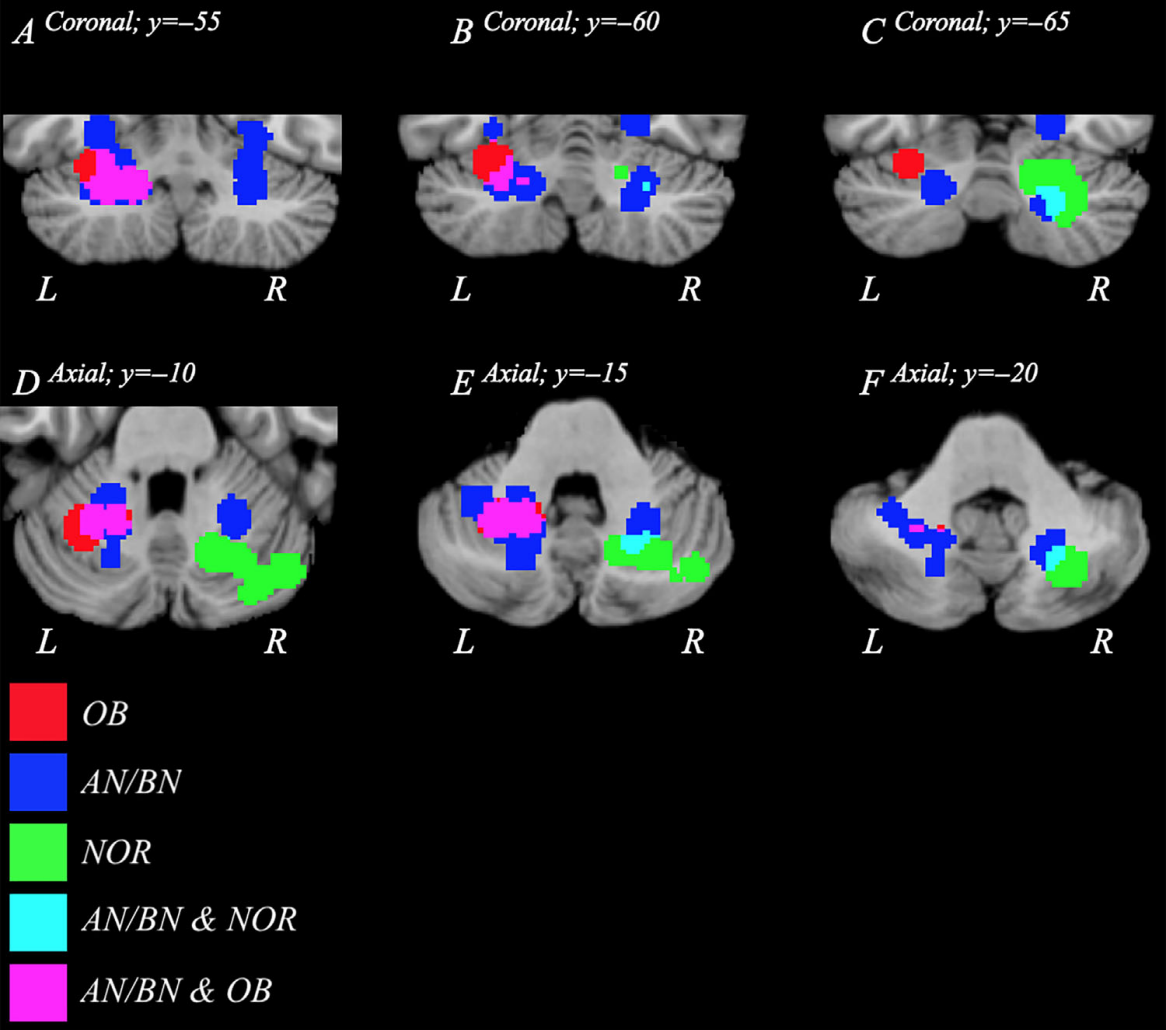
Family‐wise error (FWE)‐corrected overlay clusters visualizing decreases in volume from AN/BN (blue), OB (red), and NOR (green) data. Regions of the cerebellum where AN/BN and OB data overlap are shown in purple, while an overlap in AN/BN and NOR data is displayed in cyan. No overlap between OB and NOR data was present. Panels A–C and D–F depict the logical overlays in axial and coronal orientations, respectively. AN, anorexia nervosa; BN, bulimia nervosa; L, left; NOR, normative; OB, obesity; R, right.

Pooling combinations of data (AN/BN‐OB, AN/BN‐NOR, OB‐NOR) showed that cerebellar volume reduction significantly overlapped across cohorts, but these reductions were also largely distinct according to body weight condition as well as across BMI (Figure S2). OB and NOR clusters were exclusive to the left and right hemispheres, respectively, while AN/BN clusters were bilaterally located. Logical overlays on *MANGO* delineated significant intercondition overlap, which prompted three conjunction analyses (Figure 5). The AN/BN–OB conjunction analysis revealed a large region of overlap. The cluster was located in the left Lobule VI with a volume of 0.214 cm^3^ and center coordinates of −24, −55, −33 (Figure 6.1; Table 5). Conjunction analyses between clinical cohorts and the NOR cohort conducting correlational analyses between volume and BMI revealed additional findings. The AN/BN–NOR conjunction analysis revealed a unilateral overlap of structural reduction. The cluster was 0.0296 cm^3^ with a cluster center of 23, −64, −39, and primarily located within right Lobule VI, but also affected the surrounding Crus II and dentate nucleus regions (Figure 6.2; Table 5). No overlap or combinatorial clusters falling within our significance threshold were found in the OB–NOR conjunction analysis.

**FIGURE 6:**
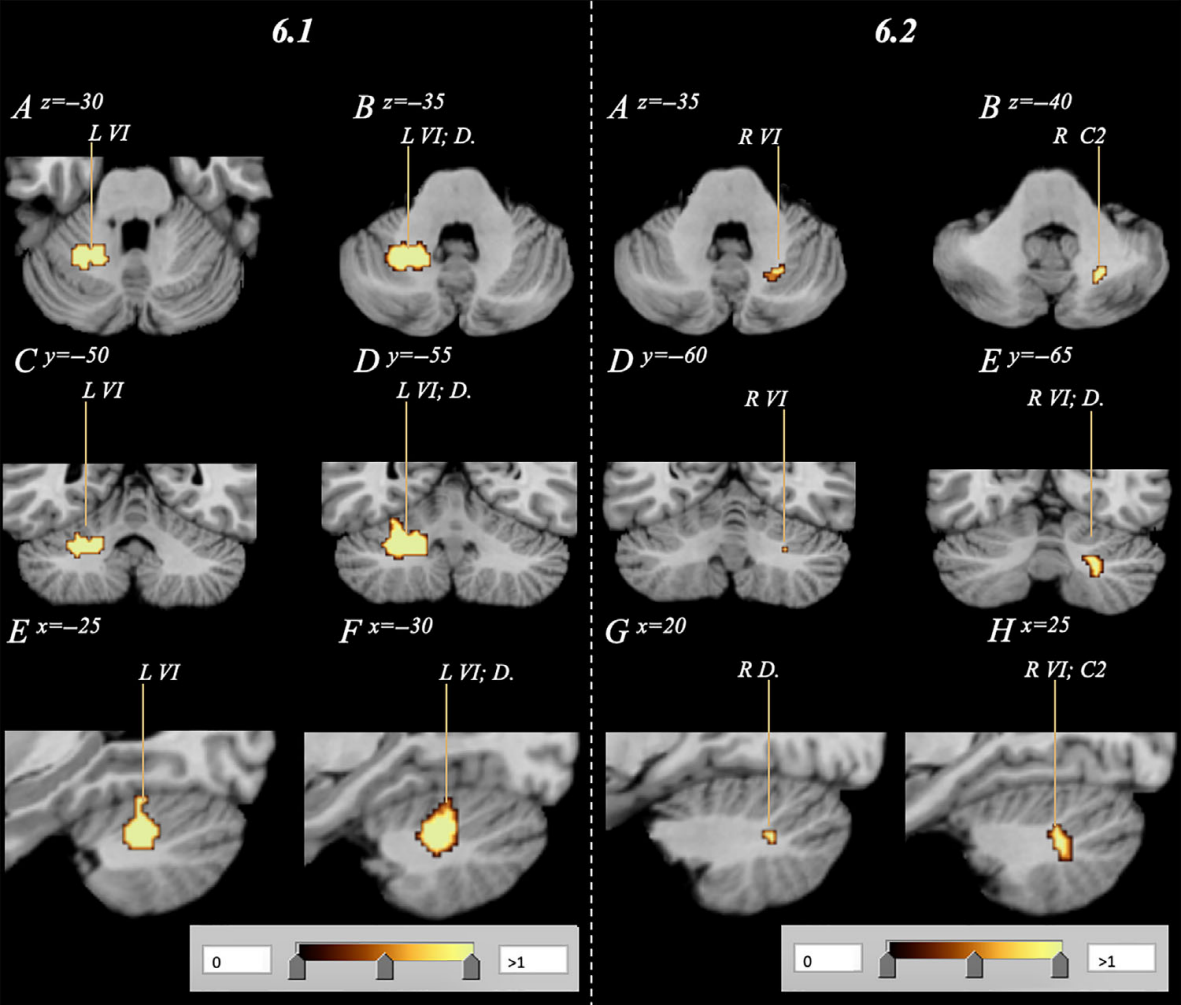
Subsequent conjunction analysis combining respective AN/BN and OB data (6.1) as well as AN/BN and NOR data (6.2) to visualize affected regions of overlap. Analyses confirm an overlap in volumetric decrease in both AN/BN and OB subjects, as well as AN/BN and NOR subjects primarily within Lobule VI (*p* < .05). Subfigures 6A/B, C/D, and D/E depict the combinatorial clusters in axial, coronal, and sagittal orientations, respectively. No overlap between OB and NOR data was identified. AN, anorexia nervosa; BN, bulimia nervosa; C2, Crus 2; D., Dentate; NOR, normative; OB, obesity; VI, Lobule 6.

**TABLE 5:** Conjunction analysis clusters of significance in AN/BN, NOR, and OB cohorts.

Study	MNI coordinates			Volume (cm^3^)	Volume breakdown (%)	ALE score
	*x*	*y*	*z*			
(AN/BN–OB)						
L CB	–24	–55	–33	0.214	54.9% Ant.; 45.1% Post.	.00850
L CB, posterior	–28	–54	–34			.00854
L CB, posterior	–18	–56	–34			.00768
(AN/BN–NOR)						
R CB	23	–64	–39	0.029	100% Post.	.00690
R CB, posterior	24	–64	–40			.00689
(OB–NOR)						
NS						

Abbreviations: ALE, activation likelihood estimation; AN, anorexia nervosa; Ant., anterior lobe; BN, bulimia nervosa; CB, cerebellum; L, left; NOR, normative; NS, not significant; OB, obesity; Post., posterior lobe; R, right.

### 3.5 Sensitivity analyses

Sensitivity analyses were conducted on all available cohort data sets (Table S2). One‐by‐one omission of publications identified heterogeneity across OB findings, in which the left posterior and anterior cerebellar clusters were present in three of six analyses. In contrast, NOR and AN/BN findings were robust, with the right posterior cerebellar cluster demonstrating repeatability in 10 of 10 NOR analyses, as well as left and right AN/BN cerebellar clusters appearing in 11 of 11 and nine of 11 analyses, respectively. While the AN‐only cohort did not report findings, the jackknife analysis identified the left posterior CB as reduced upon removal of Lenhart et al. (2022), Phillipou et al. (2018), Fonville et al. (2014), and Bomba et al. (2015).

## 4 DISCUSSION

### 4.1 Cerebellar differences: OB versus AN/BN versus HC

Compiling data from OB and AN/BN publications, as well as from correlations between BMI and brain volume, confirm cerebellar structure to be significantly decreased within varying BMI states and on extremes of the spectrum of body weight disorders. However, we are unable to conclude that cerebellar alterations occur in AN alone, with existing differences between AN‐only and AN/BN cohorts potentially due to global atrophy of gray matter associated with AN‐predominant weight loss. As such, subsequent interpretation of identified findings and potential structural mechanisms will focus on the combinatorial appetitive dysregulation presented by the AN/BN cohort. From cohort data, regions Crus I and Lobule VI were most consistently and significantly affected, identifying two cerebellar regions with respective executive and sensorimotor functionality associated with conditions of dysregulated BMI. In addition, findings indicate both condition‐specific differences as well as significant overlap in cerebellar GMV reduction between AN/BN, OB, and NOR cohorts. Reduced GMV in Lobule VI, Crus I, and the dentate nucleus was present in AN/BN, OB, and NOR cohorts but differed according to hemisphere. In the NOR population, affected regions correlating with increased BMI overlapped with cerebellar reduction seen in AN/BN, but not the OB analysis. Differential hemispheric involvement of two regions that reportedly comprise distinct corticocerebellar circuits leads us to suggest that multiple cerebellar regions are reliably and consistently involved across states of appetite dysfunction. Understanding of these regional functions may provide insight as to how the cerebellum may differentially contribute to the dysregulation of body weight.

Crus I, structurally reduced in AN/BN, OB, and NOR cohorts, is reported to play predominant executive‐, memory‐, and some emotional‐related functions. Crus I and II contain specific functional localization that corresponds to cerebral cortical zones (Edge et al., 2003; Guell et al., 2018; Habas et al., 2009; Heinitz et al., 2017; Marron et al., 2019; Stoodley & Schmahmann, 2009) and falls within the ECN (Habas et al., 2009; Stoodley, 2012; Stoodley & Schmahmann, 2009). In typical populations, this network plays roles in executive function, spatial attention (Ciricugno et al., 2020), and verbal working memory (Edge et al., 2003; Guell et al., 2018; Iglói et al., 2015; Shen et al., 2020; Stoodley & Schmahmann, 2009), as well as goal‐directed behavior via communication with the hippocampus (Iglói et al., 2015). Recently, Crus II is reported to serve emotional self‐experience and social mentalizing (Van Overwalle et al., 2020), expanding emotional roles played by posterior cerebellar lobes.

Functional hemispheric differences reported in Crus I may explain how the cerebellum is implicated, but likely plays distinct roles in cases of pathological under‐ and overeating. Bilateral cerebellar reduction has previously been associated in those with AN (Zhang et al., 2018). Despite no cluster‐based findings within the exploratory AN‐only cohort, Crus I reduction within the AN/BN cohort may be associated with general executive and attentional deficits reported in undereating (Gaudio et al., 2018), such as body image disturbance and behavioral rigidity toward food. Alternatively, reduction of Crus I within this cohort may be more strongly associated with BN‐specific symptomatology, such as altered traits of impulsivity or LOC eating. Additionally, volumetric reduction of Crus II was only identified within the NOR and AN/BN findings, suggesting socioemotional functions of Crus II could play a less prominent role in those with OB. Crus I reduction was specific to the left hemisphere in the OB cohort, which interestingly overlaps with previous unilateral cerebellar PSE (English et al., 2019) findings as well as regions activated in spatial attention (Ciricugno et al., 2020) and social processing tasks (Guell et al., 2018). Thus, cerebellar volume reduction in cases of overeating or OB seen in this meta‐analysis may suggest altered measures of food‐related attention, impulsivity, working memory, or loss of control, aspects characteristic of OB (Coppin et al., 2014; Cortese & Vincenzi, 2011; Cortese et al., 2016; Pineda‐Alhucema et al., 2018; Wu et al., 2017; Yang et al., 2018; Zhang et al., 2018). However, sensitivity analyses reported heterogeneity among OB study findings, likely due to lacking study power from limited literature. Further region‐of‐interest structural studies as well as resting‐state and fMRI tasks relating to impulsivity, working memory, and decision making would aid in furthering condition‐specific interpretations from this work.

Within NOR populations, reduction of Crus I was associated with increased BMI, but reduction was unilateral to the right hemisphere and included reduction of Crus II, suggesting that differential mechanisms may underly distinct contributions toward weight gain. However, it is important to note the differential study power between cohorts, with NOR findings derived from a significantly larger sample size than in those with OB. While findings corroborate concepts that the cerebellum is multimodally associated with differing aspects of BMI increase, such as homeostatic‐ versus nonhomeostatic‐associated weight gain, future evaluation of NOR populations excluding those with OB would further solidify interpretations.

Lobule VI consistently reported with structural reduction in AN/BN, OB, and NOR cohorts, and its known functional distinction from Crus I furthers the concept that more than one region of the cerebellum participates in aspects of the dysregulation of body weight. Lobule VI has recently been recognized as serving several cognitive and emotional functions, and plays roles in the salience (Cacciola et al., 2017; Habas et al., 2009; Seeley et al., 2007; Stoodley & Schmahmann, 2009, 2010), somatomotor, ventral attention, and visual networks (Van Overwalle et al., 2020). Roles of the salience network, predominantly represented via Lobule VI, involve autonomic and interoceptive processing in response to various forms of salience such as emotion, reward, and homeostatic regulation (Craig, 2002; Critchley et al., 2004; Eisenberger et al., 2003). Tractography studies demonstrate connections between the motor cortex, Lobule VI, and dentate nucleus (Habas & Manto, 2018; Kelly & Strick, 2003), and fMRI studies report consistent Lobule VI activity in emotional, social, and environmental learning tasks (Bermpohl et al., 2006; Olivo et al., 2018; Takahashi et al., 2004). Computational models also support that circuits implicated in Lobule VI are important for Pavlovian conditioning and emotional learning (Adams et al., 2013; Barrett et al., 1708; Brown et al., 2013; Pezzulo, 2012; Pu et al., 2020), including perceptual inferences such as startle responses and spontaneous behavior (Friston & Herreros, 2016). Such mechanisms may contribute to AN or BN via development of food aversion or negative food/weight associations that tend to exacerbate low dietary intake or restriction. Alternatively, food may be conditioned as an excessively rewarding or positive stimulus, which may be associated with overeating or LOC consumption.

Similar to Crus I findings, differential hemispheric reduction of Lobule VI across cohorts suggests differential cerebellar involvement. While the exploratory AN‐only analysis identified no clusters of interest, the AN/BN analysis identified bilateral reduction of Lobule VI. Those with AN have previously demonstrated reduced bilateral volume of the mid‐posterior cerebellum, which is suggested to contribute toward characteristic AN symptoms such as food aversion (Zhang et al., 2018), but further functional studies focusing on the cerebellum are warranted to further understand this relationship, as well as additional studies are needed to distinguish between AN and BN findings. Reduction of Lobule VI in OB was restricted to the left hemisphere, which contains regions specific to social processing tasks (Guell et al., 2018). In contrast to the OB cohort, reduction of Lobule VI volume within the NOR population was specific to the right hemisphere. While there is currently no specific ascribed role to the right Lobule VI, differing hemisphericity of effect between OB and NOR cohorts similarly suggests differential cerebellar participation in pathological/nonpathological overeating.

Reduction of the dentate nucleus was also seen in AN/BN, OB, and NOR cohorts. This region is highly connected to the hypothalamus and thought to interact with the lateral hypothalamic area, ventromedial nucleus, dorsomedial nucleus, and paraventricular nucleus to modulate forelimb movements in food grasping behavior (Martin et al., 2000). As with previous findings, the recruitment of the dentate nucleus within multiple cohorts not only suggests cerebellar implication in both nonpathological and clinical states of body weight dysregulation, which may functionally differ due to associations with opposing hemispheres, but also that multiple cerebellar regions are associated with the dysregulation of body weight and BMI.

Lastly, a lack of cluster‐based findings within the AN‐only analysis contrast previous reports of cerebellar atrophy in AN (Boghi et al., 2011; Seitz et al., 2018; Zhang et al., 2018) and its structural susceptibility to starvation (Boghi et al., 2011), and do not align with reports of whole‐brain reduction in those with AN (Amianto, D'Agata, et al., 2013; Fonville et al., 2014). Findings from this study suggest aforementioned structural cerebellar contributions underlying AN pathophysiology are unlikely to be distinct to cerebellar subregions and may be partially or fully consequent of whole‐brain reduction characteristic of starvation.

### 4.2 Cerebellar overlap: OB versus AN/BN versus HC

Volumetric overlap among AN/BN, OB, and NOR cohorts revealed that cases of dysregulated appetite across the BMI‐spectrum exhibit significant reduction in similar cerebellar regions, which have been known for distinct sensorimotor (Lobule VI) and executive (Crus I/II) roles and suggest multimodal cerebellar association across the BMI spectrum. Structural reduction in cases of AN/BN and OB, as well as within NOR populations assessing cerebellar volume alongside increased BMI, was predominant to Lobule VI and Crus I. Findings relating to nonpathological BMI increase via the NOR cohort as well as the AN/BN cohort overlapped within the right Lobule VI, Crus II, and dentate nucleus. Alternatively, Lobule VI and Crus I were both reduced in OB and NOR data sets, but difference of effect according to hemisphere meant no overlap in volume reduction was identified. The implication of similar cerebellar regions yet differing hemisphericity of effect again suggests that while these cerebellar regions participate in differing forms of body weight states, the main sources of variation among NOR populations may be quite different to those driving differences in clinical conditions.

### 4.3 Limitations

#### 4.3.1 Number of studies

Our review was limited by low study numbers used for some individual analyses, and we were unable to provide sufficient information on race/ethnicity and socioeconomic status. Availability of data also prevented further evaluation into sex, age, hormonal differences, and brain inflammation values across participants. Due to such limitations, we confined our analysis to changes in GMV. Future research would benefit from utilizing more specified imaging technology catered to WMV alterations, such as diffusion tensor imaging and diffusion‐weighted imaging.

Similarly, while there is no definitive instruction on the minimum number of studies needed to conduct an ALE meta‐analysis, the GingerALE (2.0) manual mentions that a minimum of 20–30 coordinates per experiment, as seen in our analyses, is sufficient to produce significant and valid clusters for simple paradigms. Later on, Eickhoff et al. (2016) recommended approximately 15–17 studies for reliability of analysis results, which were not present in our study. While cohort‐specific sensitivity analyses were implemented to attempt to account for heterogeneity across findings, little can be done to correct or adjust for small sample sizes via GingerALE. A method of counteracting potentially unreliable results from future smaller studies would be to analyze small‐cohort findings alongside larger data sets, or to incorporate quantifications of bias from small sample sizes (i.e., Egger test) within GingerALE software utility.

#### 4.3.2 Assumptions

The research question posed by this review—to determine if areas of the cerebellum involved in different disorders of appetite control were the same or distinct, was driven by a review of the literature providing extensive evidence that the cerebellum is implicated in disorders of appetite control (see Section 1). Therefore, our study is based on this assumption and does not independently verify it. This is because we only reviewed studies that included cerebellum findings rather than all MRI studies of eating disorders. Additionally, both AN/BN and OB are associated with elevated inflammation within the brain (Seitz et al., 2019; Spyridaki et al., 2016), which may influence findings related to volumetric reduction of the cerebellum. Future studies would benefit from including brain inflammation factors within analysis.

#### 4.3.3 Causality and direction of effects

Further, we are unable to verify the direction of causation in found associations. A common limitation in neuroimaging studies is their capacity to determine causality. The studies reviewed here are only able to demonstrate associations between volume abnormalities and condition, and it is unclear whether reduced volumes are a cause or subsequent effect.

#### 4.3.4 Bulimia nervosa

Although we excluded the majority of BN patients, approximately 20% of clinical AN data consisted of those with BN that we were unable to remove. As there were insufficient BN papers (*n* = 3) to conduct individualized cohort analyses for AN and BN, data sets were thus pooled into one AN/BN cohort. While there is a significant overlap in comorbidity and symptomatology between those with AN and BN (D'Agata et al., 2015), including this group may have resulted in volume reduction not linked to states relating to those with AN, and findings from this meta‐analysis are unable to distinguish between those with mixed AN/BN pathology and those with AN alone. We attempted to mitigate limitations regarding AN/BN etiological heterogeneity by conducting an exploratory assessment with AN‐only publications. Nevertheless, our study still focuses on reduced cerebellar volume in eating disorders, which is clearly the case for bulimic individuals.

## 5 CONCLUSION

While theories proposing cerebellar functions in emotional, cognition, and conditioning behaviors are now receiving wide acceptance, a role in weight and appetite is rarely discussed. In this review, we found utility in exploring cerebellar differences and similarities in states at opposite ends of the body weight dimension, and collated structural evidence from many sources. Altogether, results of our ALE analyses support the concept that the cerebellum is associated with dysregulated appetite, eating disorders, and body weight in both pathological and nonpathological states. We found that while cerebellar associations with bodyweight issues recruited similar regions, hemispherical effects differed according to the type of appetitive condition, suggesting different cerebellar circuitry contributing to eating behavior between nonclinical and pathological populations. Such findings add to a body of evidence theorizing cerebellar participation in appetite‐ and feeding‐related domains. Utilizing our knowledge of the emotional and cognitive functions of the cerebellum will assist in identifying novel remedial approaches to manage disorders of appetite regulation as well as increase efficacy of interventions provided to clinical populations.

## AUTHOR CONTRIBUTIONS

**Michelle Sader**: Conceptualization; methodology; investigation; writing—review and editing. **Gordon Waiter**: Study visualization; methodology; resources; writing—review and editing; supervision. **Justin Williams**: Conceptualization; study visualization; methodology; writing—review and editing; supervision.

## CONFLICT OF INTEREST STATEMENT

The authors declare no conflicts of interest.

### PEER REVIEW

The peer review history for this article is available at https://publons.com/publon/10.1002/brb3.3286.

## Supporting information

Supporting Information

## Data Availability

Additional data supporting study findings are available from the corresponding author upon request.
